# The effects of visual working memory load on detection and neural processing of task-unrelated auditory stimuli

**DOI:** 10.1038/s41598-023-31132-7

**Published:** 2023-03-16

**Authors:** Laura Brockhoff, Laura Vetter, Maximilian Bruchmann, Sebastian Schindler, Robert Moeck, Thomas Straube

**Affiliations:** 1 Institute of Medical Psychology and Systems Neuroscience, University of Muenster, Von-Esmarch-Str. 52, 48149 Münster, Germany; 2 Otto Creutzfeldt Center for Cognitive and Behavioral Neuroscience, University of Muenster, Münster, Germany

**Keywords:** Cognitive neuroscience, Attention

## Abstract

While perceptual load has been proposed to reduce the processing of task-unrelated stimuli, theoretical arguments and empirical findings for other forms of task load are inconclusive. Here, we systematically investigated the detection and neural processing of auditory stimuli varying in stimulus intensity during a stimuli-unrelated visual working memory task alternating between low and high load. We found, depending on stimulus strength, decreased stimulus detection and reduced P3, but unaffected N1 amplitudes of the event-related potential to auditory stimuli under high as compared to low load. In contrast, load independent awareness effects were observed during both early (N1) and late (P3) time windows. Findings suggest a late neural effect of visual working memory load on auditory stimuli leading to lower probability of reported awareness of these stimuli.

## Introduction

*Load Theory*^1–3^ can be regarded as the dominant theory of how attention influences information processing. It proposes a flexible locus of attention depending on the load of an ongoing task. During low load, information can be processed, while high load leads to the early inhibition of distractor processing. Initially, the theory was developed in the context of perceptual task manipulations (i.e., perceptual load)^1,2^. Later versions also included so-called cognitive load (i.e., working memory load)^4^. Originally, the theory suggested opposite phenomena for perceptual and working memory load^4^. However, this assumption was later revised^5^, and empirical work shows similar effects of perceptual and working memory load (for review, see^6^).

*Load Theory* suggests early effects of high versus low load on the processing of task-unrelated distractors^3^. However, what constitutes early and late effects in neuroscientific terms remains vague but can broadly be operationalized by early versus late brain areas in the processing hierarchy and specific temporal windows in electrophysiological responses^6^. A recent review of our group^6^ regarding load effects on neuronal activation to task-unrelated distractors led to the conclusion that load effects occur more reliably in later stages of stimulus processing, but that the temporal occurrence of load effects remains to be specified. Electrophysiological methods offer a high temporal resolution of neuronal activity and allow for differentiation between early and late effects^7–10^. In the auditory modality, the N1, for example, is an early ERP component occurring around 100 ms after stimulus onset and representing the earliest negative component associated with changes in the auditory environment^11^. In contrast, late positive waves from 300 ms post-stimulus onwards are less dependent on stimulus features but associated with post-perceptual processing of stimuli, including decision making and evaluation of stimuli^12,13^.

Several studies investigated neural correlates of working memory load on task-unrelated stimuli using electroencephalography and yielded heterogeneous findings. In several visual unimodal studies (i.e., visual load with a visual distractor), early visual potentials (i.e., P1 and N1/N170 amplitude) were reported to be uninfluenced by load^14–16^, while two studies reported decreased face-related N170 amplitudes under high load^17,18^. Later visual ERP amplitudes (i.e., P3 amplitude) are systematically decreased in these studies^14–19^. In the auditory modality, there are no unimodal studies. For crossmodal auditory distractors (i.e., visual load with an auditory distractor), some studies report decreased N1 amplitudes^20,21^, while other studies find no effects^22^. Here, later ERP amplitudes (i.e., P3 amplitude) to auditory distractors are also systematically decreased^20,22–24^, with only one study showing increased P3 responses^25^. Thus, despite studies suggesting that working memory storage is domain-specific^26^, a large body of research shows crossmodal effects of working memory load on ERPs to auditory distractors (for review, see^6^). This speaks for crossmodal sharing of processing resources in accordance with the broad formulations of *Load Theory* (for review, see^6^).

Furthermore, a critical prediction from *Load Theory* is that the detection of distractor stimuli, at least threshold stimuli, should be reduced or even inhibited under conditions of high load. However, there is only one study that investigated detection and neural processing of distractors depending on the load level of the main task. Molloy et al.^27^ found reduced magnetoencephalographic early (aM100) and late (P3) responses to sounds under high versus low perceptual visual load and reduced detection of sounds under high compared to low load. This suggests that decreased neural responses are associated with the lower awareness of the sounds. However, no study has investigated whether working memory load affects the detection of distractors. Therefore, it remains unclear whether there are effects of working memory load on the detection of distractors, whether this depends on stimulus strength of distractors and whether load effects are seen in parallel in early and late neural responses and behavioral data. Furthermore, in most studies, load effects on ERPs to distractors are difficult to delineate from load effects of the main task. Suited control conditions, such as load variations without any stimulus, are necessary (for extended discussion, see^4^).

In the current study, we systematically investigated the effects of visual working memory load on the detection and neural processing of auditory distractors. To better understand the role of stimulus strength, we varied the intensity of auditory distractors. Furthermore, we compared conditions in which a distractor or no distractor was presented to separate distractor and general load effects. We used ERPs to delineate early and late effects of load on neural responses to distractors. On the behavioural level, the detection rate of auditory distractors was hypothesized to increase with sound intensity. However, detection of auditory stimuli should be reduced under high compared to low working memory load, especially near detection threshold. For the ERP, we hypothesized an increase of the N1 amplitude with sound intensity and a reduction in amplitude by increased working memory load. Regarding the P3, we also hypothesized an increase in amplitude with sound intensity and a reduction in amplitude by increased working memory load. In both cases, we hypothesized that load effects are most pronounced for stimuli near detection threshold. The pattern of load effects was assumed to be similar to load-independent effects of stimulus awareness, which are known to be associated with reduced amplitudes in early and late time windows for unaware as compared to aware auditory stimuli during detection tasks^28–30^.

## Methods

### Participants

Fifty-one participants were recruited at the University of Muenster. One participant aborted the study, and seven participants were rejected due to excessive EEG artifacts, leading to a final sample size of 43 participants (14 males, 29 females; M_age_ = 24.56 years, SD_age_ = 3.671 years). They gave written informed consent and received 10 Euros per hour for their participation. All participants had normal or corrected-to-normal vision and audition and no neurological or psychiatric disorders history. Except for one, all participants were right-handed. The study was approved by the medical ethics committee of University of Muenster, and all procedures were carried out in accordance with this declaration.

### Stimuli

An iiyama GMaster GB2488HSU monitor (iiyama, Nagano, Japan) at 60 Hz with a resolution of 1920 × 1080 pixels was employed for stimulus display. The viewing distance amounted to 60 cm. Visual stimuli were black-colored consonant letters (height = 2.49 degree of visual angle) on a grey background. These letters were presented equally spaced (3.23 degree in-between) next to the white fixation cross (a “+” consisting of two bars of 0.373 × 0.124 degree) in the center of the screen. Two or six letters were randomly chosen and presented in the low or high working memory load condition. Auditory stimuli comprised two components: (1) babble noise and (2) beep stimuli. The babble noise was taken from the freely available signal processing information database^31^ accessible via http://spib.linse.ufsc.br/noise.html, and validated in a study by Schlossmacher et al.^28^. The noise was further processed by adding the same stream played backward to the original. The average sound pressure level (SPL) during the experiment amounted to 55 dB. Beep stimuli were sinusoidal tones with rise and fall times of 10 ms, a duration of 100 ms, and a frequency of 600 Hz. They were presented at three different volumes (low, medium, and high intensity) and additionally with zero intensity (“no sound”), each with a proportion of 25%. In each trial, one beep stimulus (or the “no sound” stimulus) was embedded in the babble noise at a pseudo-randomized time point between 200 ms after the onset and 200 ms before the offset of the fixation cross in letter maintenance phase. The sound volume was determined in a behavioral pilot study (*n* = 10). The participants were asked to respond to a simple visual detection task (color change of a fixation cross) while the above-described auditory stimuli were played in the background. After each trial, participants were asked whether beep stimuli were perceived. The experimenter adjusted the sound volume manually until five participants detected the beep in about 50% of the trials. This volume was then multiplied by 0.5 and 2 to yield the three critical levels used in the main experiment (42 dB, 45 dB, and 48 dB). Thus, this allowed us to investigate how load findings are influenced by stimulus strength varying from below detection threshold to detection threshold and above detection threshold.

### Procedure

**Figure 1:**
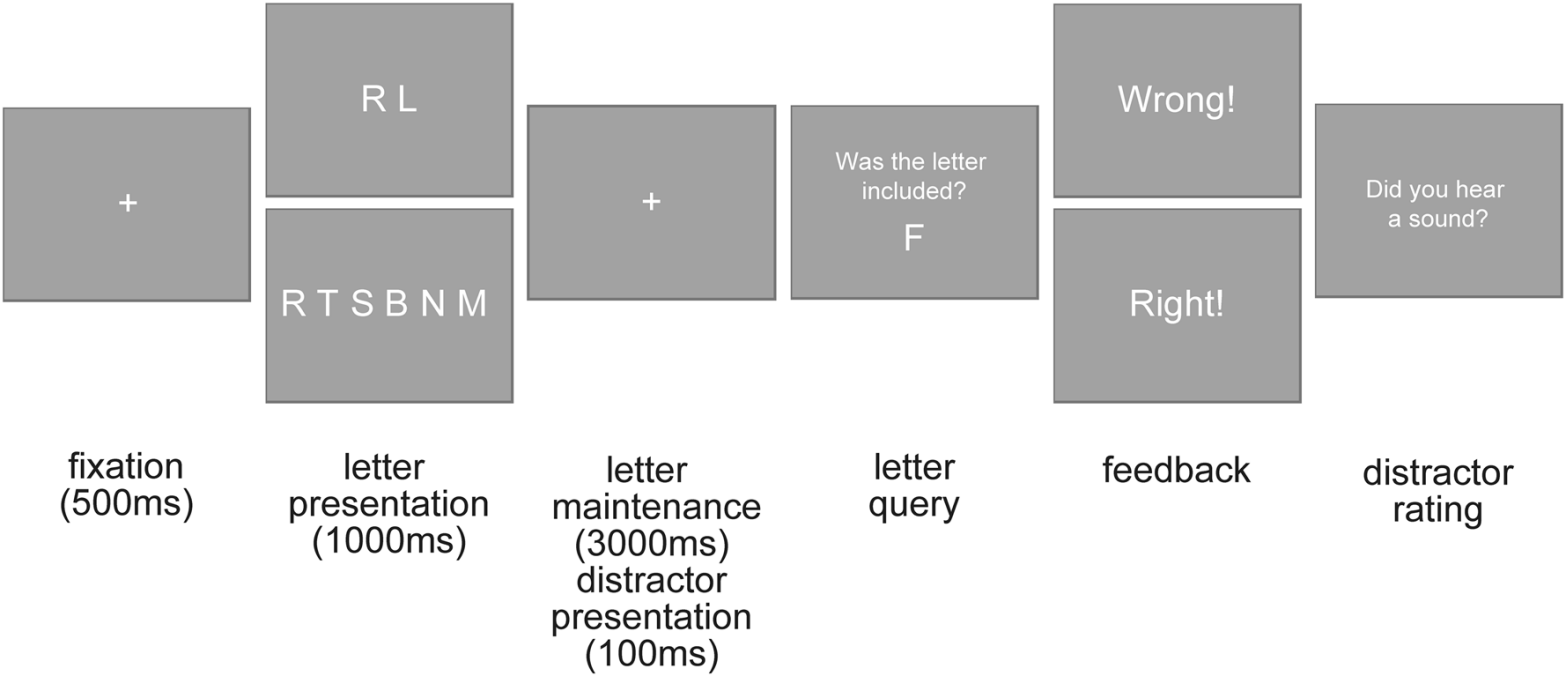
Illustration of the experimental course.

Participants first responded to a demographic questionnaire and were prepared for the EEG. Afterward, they completed a practice task in which they were instructed to memorize the presented letters. Moreover, they were told that the background babble noise would contain occasional beeps with varying sound volume levels. The instructions were designed to establish the memory task as the main task and the report of the auditory percepts as a subordinate task. This was achieved by stating that for the memory task, they would receive or lose points for correct and incorrect answers, whereas the sounds had no relevance to the participants. Moreover, they were instructed that they would first have to respond to the memory task, then receive the point feedback, and at last would be asked to rate their performance on the sound perceptions. It was repeated that the sound rating would not lead to point win or loss. Moreover, the different query format for the visual task (i.e., explicit yes–no-query) and the sound perception (i.e., rating, see below) was also intended to emphasize the importance of the memory task. Then the main experiment started, divided into 12 blocks with self-controlled breaks with a maximum length of 3 min. Each block included 32 trials of both load levels, with trials aborted due to excessive eye movement being appended to the end of a block. A single trial (see Fig. 1) started with the presentation of the fixation cross for 500 ms. Afterward, the letters (i.e., 2 letters in the low working memory load conditions and 6 letters in the high working memory load condition) appeared for 1 s on the screen. Following this, the fixation cross was presented again. During this 3-s presentation, the background sounds were played. The query of the memorized letters followed this. For the query, one letter appeared in the middle of the screen, and the subjects had to indicate whether this letter was among the letters to be remembered or not. In 50% of the trials, this was one of the letters to be remembered. Participants received feedback for their answers concerning the letters (i.e., plus points for correct answers and minus points for incorrect answers), accumulating over the whole experiment. After the memory query, participants rated their subjective sound perception on a four-point scale, ranging from “no—certain” over “no—uncertain” and “yes—uncertain” to “yes—certain”. After each block, they were given their total score earned (i.e., You have scored x points out of a maximum y points so far).

### EEG recording and preprocessing

EEG signals were recorded from 64 BioSemi active electrodes using BioSemi's Actiview software (www.biosemi.com). Four additional electrodes measured horizontal and vertical eye movement. The recording sampling rate was 512 Hz. The software BESA research (Version 6.0, www.besa.de) was used for preprocessing. Offline data were re-referenced to average reference and filtered with a high-pass forward filter of 0.1 (6 dB/oct) and a 30 Hz low-pass zero-phase filter (24 dB/oct). Recorded eye movements were corrected using the automatic eye-artifact correction method implemented in BESA^32^. Noisy EEG sensors were interpolated using a spline interpolation procedure. Data were segmented from 200 ms before stimulus onset until 800 ms after stimulus presentation. Baseline correction used 200 ms before stimulus onset. Data were re-referenced from the CMS/DLR to a common average reference. Trials were rejected based on an absolute threshold (> 120 µV), signal gradient (> 75 µV/δT), and low signal (i.e., the SD of the gradient, < 0.01 µV/δT). On average, 4.51 electrodes per participant were interpolated (*SD* = 2.91), and 12.58 percent of all trials per participant were rejected (*SD* = 10.57). The number of trials per load and sound volume level ranged between 27 and 49 trials (*M* = 42.48, *SD* = 5.207).

### Eye-tracking recording

We used an eye-tracker to continuously track and evaluate gaze position during the experiment, stopping experimental presentation whenever the gaze deviates more than 3° at a circular region around the fixation mark. Aborted trials were added to the end of the block. Eye-tracking was measured with the Eyelink 1000 eye-tracker from SR research (EyeLink 1000, SR Research Ltd., Mississauga, Canada). Participants were asked to place their heads on a chin rest, and the right eye was recorded. The recording sampling rate was 1000 Hz. Before each presentation block, an eye-tracker calibration procedure was automatically initiated using a five-point calibration procedure.

### Statistical analyses

Behavioral data were analyzed in MATLAB R2019b (https://de.mathworks.com). To measure the task performance, the recognition of letters was analyzed with d prime (*d*′) for the two load conditions, followed by a paired-sample *t*-test. The detection sensitivity (*aZ*) was calculated for the two load and three sound volume conditions for sound detection. *AZ* quantifies the area under the receiver operating characteristic (ROC) curve, calculated from the proportion of hits and false alarms per level of confidence^33^. It ranges between 0.5 (chance performance) and 1 (perfect detection). Sound detection data were analyzed using a Repeated Measurement Analyses of Variance (ANOVA) with load (low load (LL) vs. high load (HL)) and sound volume (low volume vs. medium volume vs. high volume) as factors.

**Figure 2:**
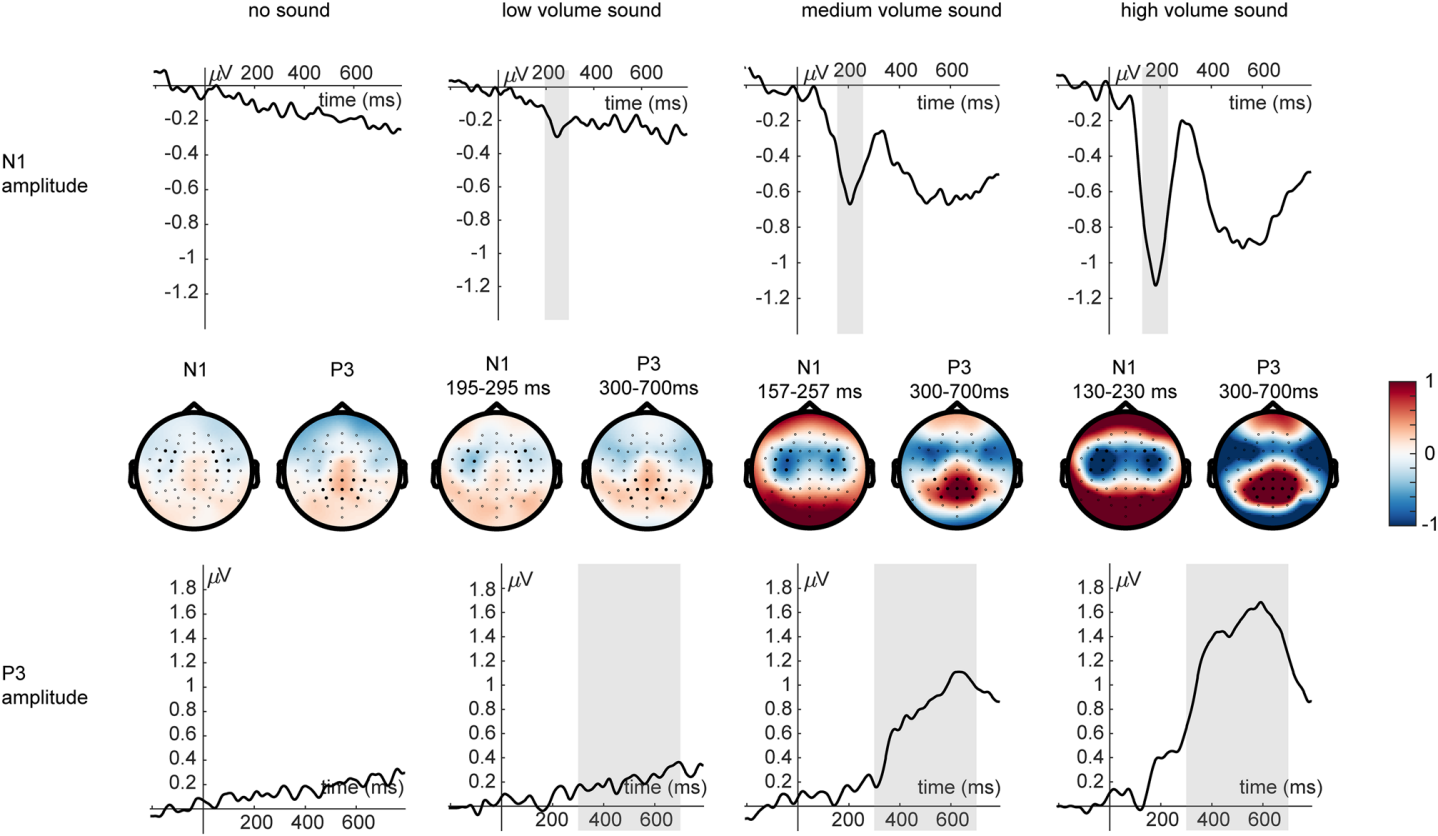
ERPs of the grand average (*n* = 43) for the two load conditions and the four sound volume conditions in the N1 time range (top row) and in the P3 time range (bottom row) with topographies (middle row).

EEG scalp data were analyzed and visualized using MATLAB R2019b (https://de.mathworks.com) and SPSS (https://www.ibm.com/de-de/analytics/spss-statistics-software). The two components of interest (N1 and P3 amplitude, see Fig. 2) were chosen as follows: We inspected the time course of topographies of the grand average for each sound level except the "no-sound" condition. Data was aggregated across all participants and averaged across the two load levels. For all sound levels above zero, we observed the typical bilateral frontal negativity peaking between 100 and 200 ms^11^. For the N1 analysis, we chose all sensors that showed the negative peak commonly at each sound level (see Fig. 2). This bilateral frontal cluster consisted of the sensors F3, F4, F5, F6, FC3, FC4, FC5, FC6, C3, C4, C5, and C6. Similarly, we identified the P3, characterized by a parieto-central positive pole typically between 300 and 700 ms^13,34^. The cluster common to all sound levels consisted of the sensors CP1, CP2, CP3, CP4, CPz, P1, P2, P3, P4, Pz, POz, PO3, and PO4. The temporal intervals of interest were identified by inspecting the ERPs averaged across the above-mentioned sensor groups, separated for each sound volume but average across load levels. For the N1 amplitude, we observed an increase in peak latency with sound volume^35,36^. Thus, the peak of the N1 amplitude for the low volume sound was identified in the time range of 195 to 295 ms, for the medium volume sound in the time range of 157 to 257 ms, and for the high volume sound in the time range of 130 to 230 ms post-stimulus. The peak of the P3 amplitude was identified for all sound volumes in the same time range of 300 to 700 ms post-stimulus. To inspect load and sound volume effects, we computed an ANOVA with load (LL vs. HL) and sound volume (low vs. medium vs. high) as the difference to the condition with no sound for the N1 and P3 amplitude. Partial eta-squared (*η*_*p*_^2^) was used to describe the effect sizes in all statistical tests.

In an additional analysis, we also compared heard and missed sounds regardless of load to test for typical signatures of auditory stimulus awareness, the so-called neural correlates of consciousness (NCC). In the auditory domain, the currently debated NCC candidates are early negativities (auditory awareness negativity, AAN^28,30^), which start in the N1 time window, and late positivities (such as the P3 amplitude^23,24^, but see^19^). For this purpose, we split the trials depending on the response to the auditory target per trial (heard: yes vs. no). To achieve the most reliable comparison of heard and unheard sounds, we chose the sound volume condition for each participant closest to perception threshold (i.e., with *aZ*, averaged across load, closest to 0.75). For the 36 participants, the selected volume level was medium volume. For three and four participants, the selected volume level was low or high volume, respectively. Response-dependent splitting of trials led to an average of 21.2 trials (SD = 11.33) across all participants and conditions. However, nine subjects were excluded from the analysis because they had one or more conditions with fewer trials than the selected cutoff value of 4, leaving a final subsample of 34 subjects. Thus, we computed an ANOVA with response (yes vs. no) and load (LL vs. HL) in the N1 (or AAN, 100–300 ms) and P3 (300–700 ms) time range for sounds with a volume close to the perception threshold. Partial eta-squared (*η*_*p*_^2^) was used to describe the effect sizes in all statistical tests.

## Results

### Ratings and behavioral results

#### Task performance

**Figure 3:**
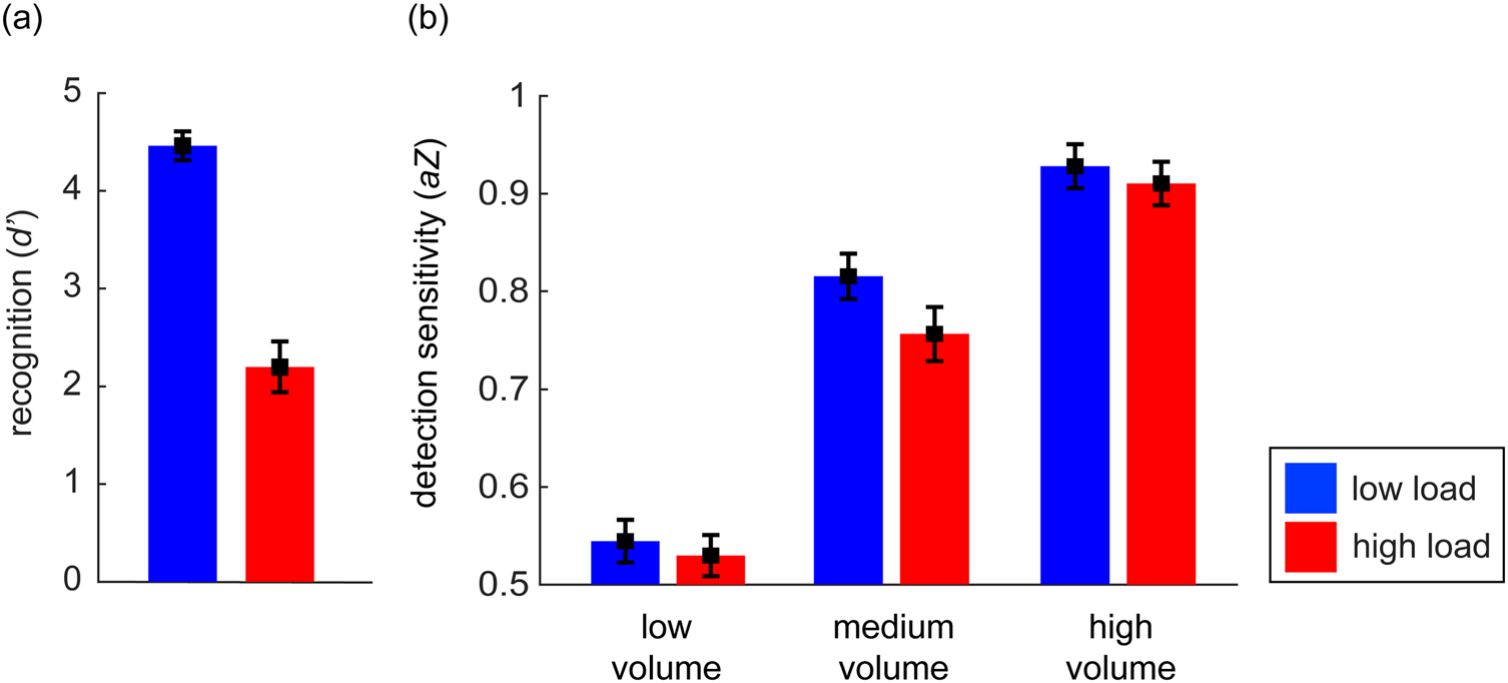
(**a**) Recognition (*d*′) in the working memory task for the two load conditions, and (**b**) detection sensitivity (*aZ*) of the sound perception for the low, medium, and high volume sound in the two load conditions.

The *d*′ was higher in the low load condition (LL, *M* = 4.47, *SD* = 0.5; Fig. 3) compared to the high load condition (HL, *M* = 2.2, *SD* = 0.87; Fig. 3). Participants were more accurate in their responses in the LL condition compared to the HL condition (*t* (42) = 17.34, *p* < 0.001; Fig. 3).

#### Sound detection

The repeated-measures ANOVA on *aZ* with load and sound volume as factors revealed a main effect of load (*F* (1,42) = 11.28, *p* < 0.01, *η*_*p*_^2^ = 0.21; Fig. 3) with lower detection sensitivity in the HL condition compared to the LL conditions. Morever, there was a main effect of sound volume (*F* (2,84) = 875.72, *p* < 0.001, *η*_*p*_^2^ = 0.95; Fig. 3) as well as an interaction of load and sound volume (*F* (2,84) = 5.64, *p* < 0.01, *η*_*p*_^2^ = 0.12; Fig. 3). For the main effect of sound volume, post hoc tests (*p* values are Bonferroni-corrected for three tests) showed that the accuracy for the sound detection increased as sound volume increased (low vs. medium volume: *t *(42) =  − 26.64, *p* < 0.001, medium vs. high volume: *t* (42) =  − 18.51, *p* < 0.001). For the interaction of load and sound volume, planned comparisons revealed that the detection of the medium volume (*t *(42) = 4.44, *p* < 0.001) and the high volume sound (*t *(42) = 2.95, *p* < 0.01) were better under LL compared to HL, while no difference was found for the low volume sound (*t *(42) = 0.58, *p* = 0.56).

**Figure 4:**
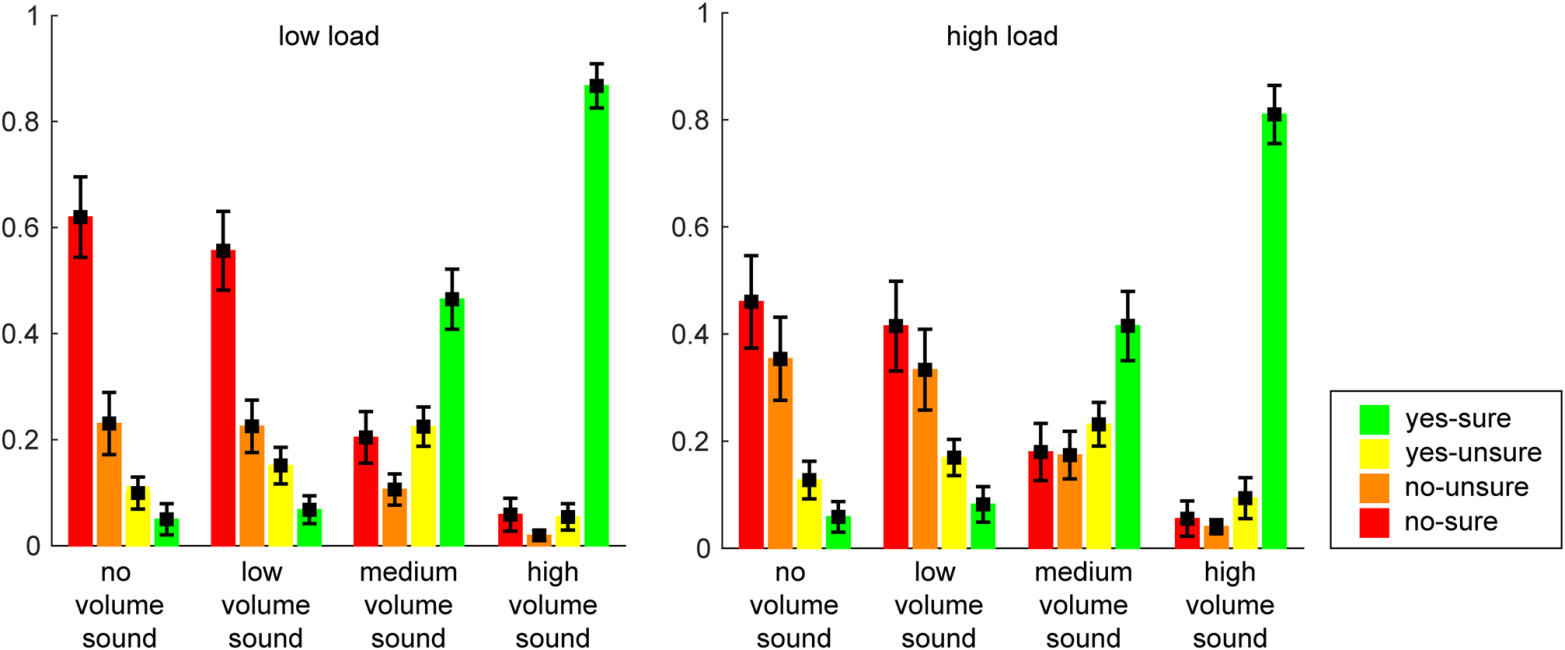
Mean distribution of the four response categories for each sound condition and the two load conditions.

**Figure 5:**
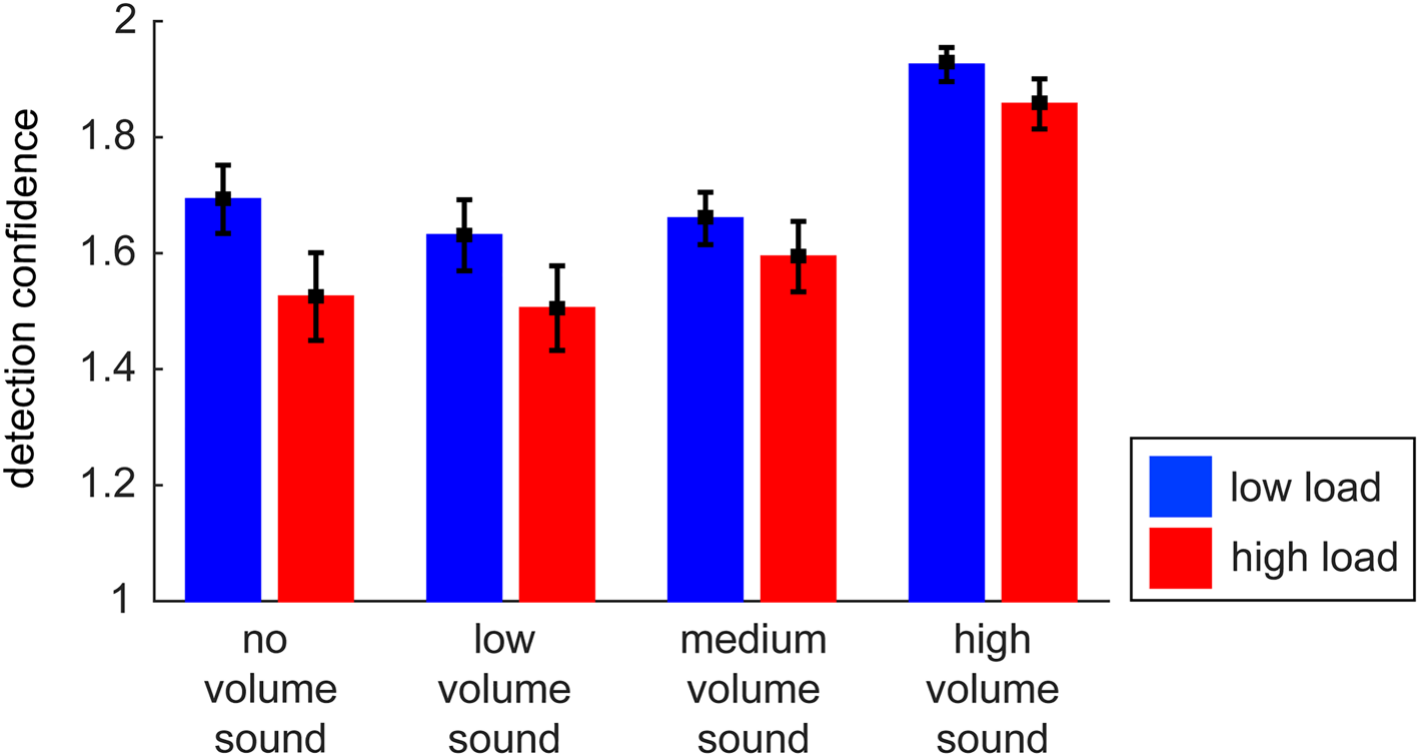
Detection confidence of the sound perception for the no, low, medium, and high volume sound in the two load conditions.

Figure 4 descriptively depicts the mean distribution of the four response categories, which are included in the calculation of *aZ*. However, visual inspection of Fig. 4 suggests that a lot of “no-sure” responses under low load turned to be “no-unsure” responses under high load for the no and low volume sound condition and thus, uncertainty seemed to be increased under high load. To complete the behavioral profiles of participants’ response behavior, we conducted an additional explorative analysis, where we compared the confidence of responses (i.e., yes-sure + no-sure responses = high confident responses (2), yes-unsure + no-unsure responses = low confident responses (1)) for each sound volume condition under the different load conditions. Note that the analyses of *aZ* and confidence are statistically not independent of each other. The repeated-measures ANOVA on response confidence with load and sound volume as factors revealed main effects of load (*F* (1,42) = 24.38, *p* < 0.001, *η*_*p*_^2^ = 0.37; Fig. 5) and sound volume (*F* (3,126) = 55.31, *p* < 0.001, *η*_*p*_^2^ = 0.57; Fig. 5) as well as an interaction of load and sound volume (*F* (3,126) = 5.84, *p* < 0.001, *η*_*p*_^2^ = 0.12; Fig. 5).

### ERPs

**Figure 6:**
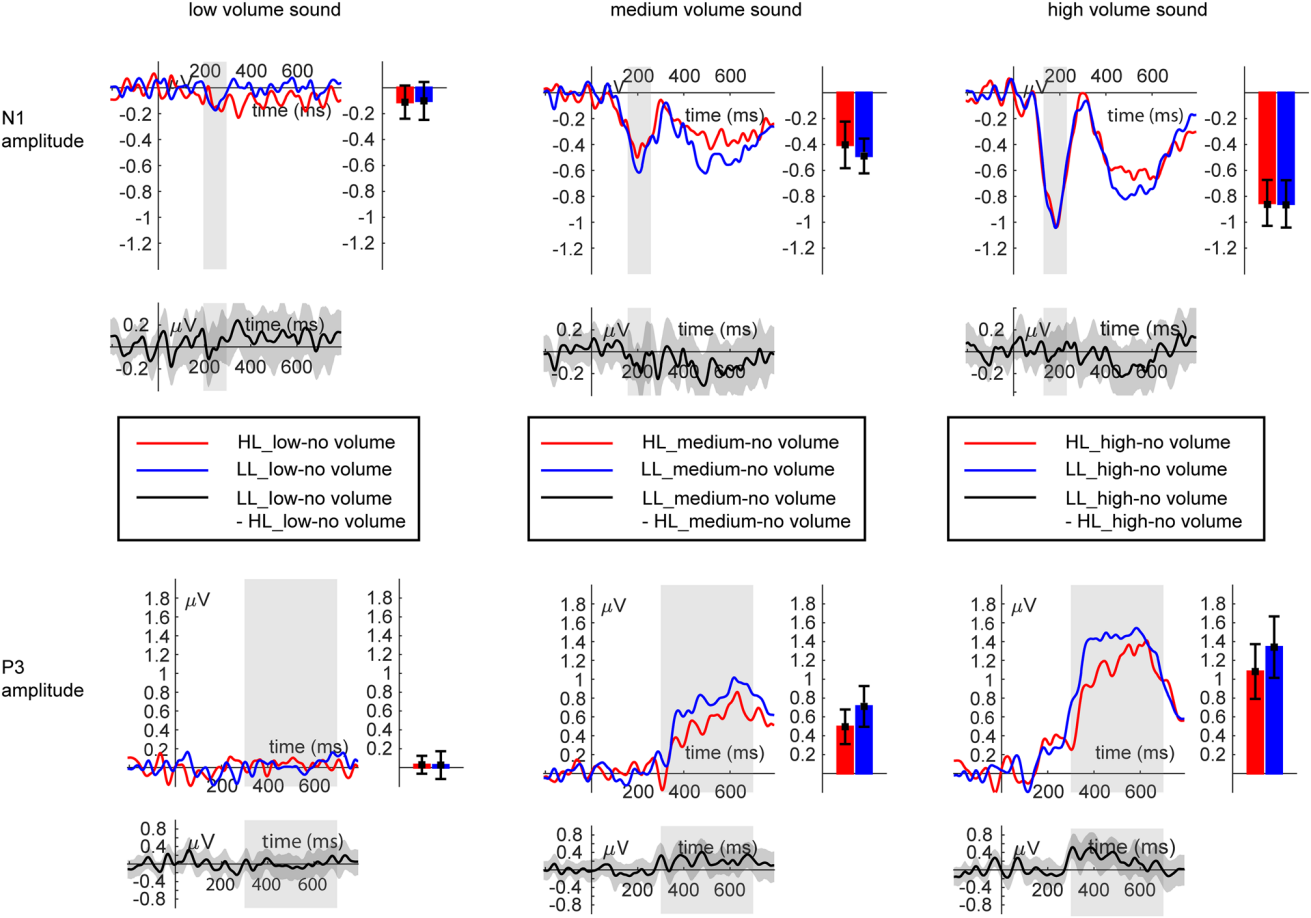
ERPs (*n* = 43) for the two load conditions (LL, HL) and the three sound volume conditions (low, medium, high) in the N1 time range (top row) and the P3 time range (bottom row).

**Figure 7:**
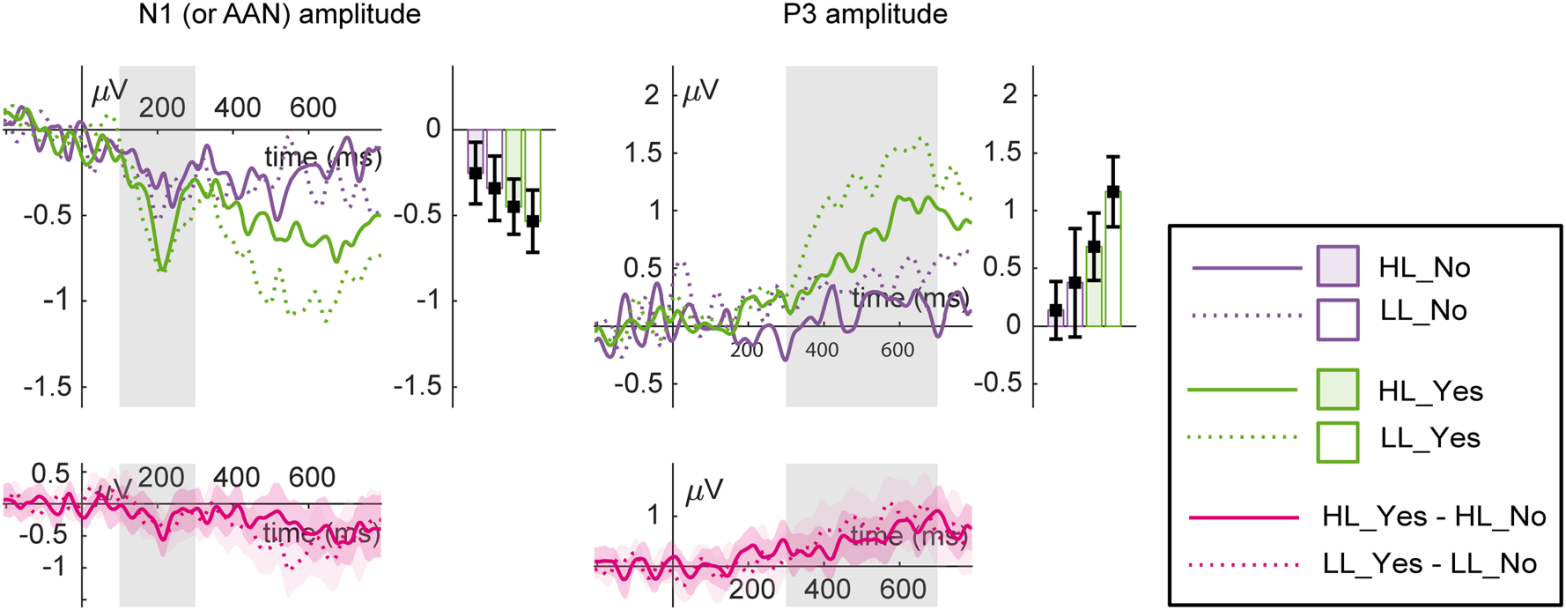
ERPs (*n* = 34) for the two report conditions (yes, no) and the two load conditions (LL, HL) in the N1 time range (left panel) and the P3 time range (right panel).

*N1* For the N1 amplitude, the main effect for sound volume was significant (*F* (2,84) = 70.56, *p* < 0.001, *η*_*p*_^2^ = 0.63, Fig. 6). Planned comparisons revealed that the N1 amplitude was significantly higher for the medium versus low volume sound difference (*t* (42) = 5.81, *p* < 0.001) and the high versus medium volume sound difference (*t* (42) = 7.62, *p* < 0.001). The main effect for load and the interaction of load and sound volume were not significant (all *p* ≥ 0.53). The additional analysis for heard vs. missed sounds showed a significant main effect of report (*F* (1,33) = 7.06, *p* < 0.05, *η*_*p*_^2^ = 0.18, Fig. 7) with higher N1 amplitudes for heard vs. missed sounds. The main effect of load and the interaction of load and report remained insignificant (all *p* ≥ 0.32).

*P3* For the P3 amplitude, the main effects for load (*F* (1,42) = 3.14, *p* < 0.1, *η*_*p*_^2^ = 0.07, Fig. 6) and sound volume (*F *(2,84) = 52.97, *p* < 0.001, *η*_*p*_^2^ = 0.56, Fig. 5) were significant. Figure 6 illustrates that the P3 amplitude is higher under LL than HL. For the main effect of sound volume, planned comparisons revealed that the P3 amplitude was significantly higher for the medium versus low volume sound difference (*t* (42) = − 6.63, *p* < 0.001) and the high versus medium volume sound difference (*t* (42) = − 6.07, *p* < 0.001). The interaction of load and sound volume was significant as well (*F* (2,84) = 2.89, *p* < 0.1, *η*_*p*_^2^ = 0.06, Fig. 6). For the interaction of load and sound volume, planned comparisons revealed that the P3 amplitude was decreased under high compared to low load for the medium volume (*t* (42) = − 1.88, *p* < 0.05) and high volume (*t *(42) = − 1.94, *p* < 0.05), but not for the low volume sound difference (*t* (42) = 0.04, *p* > 0.48). The additional analysis for heard vs. missed sounds showed a significant main effect of report (*F* (1,33) = 17.73, *p* < 0.001, *η*_*p*_^2^ = 0.35, Fig. 7) and load (*F* (1,33) = 4.84, *p* < 0.05, *η*_*p*_^2^ = 0.13; Fig. 7) with higher P3 amplitudes for heard vs. missed sounds. There was no significant interaction of load and report (*p* = 0.45).

## Discussion

This study aimed to examine the effects of visual working memory load on auditory distractor processing. We found (1) a better sound detection sensitivity under low compared to high load for the middle and high volume sound, (2) a higher response confidence under low compared to high load for each sound condition, (3) a load independent N1 amplitude for all sound volumes, (4) a decreased P3 amplitude under high compared to low load for the middle and high volume sound, and (5) load independent awareness effects during both early (N1) and late (P3) time windows.

The behavioral data shows reduced detection and lower response confidence for sounds under high compared to low working memory load. This is similar to the modulation of the detection of sound depending on visual perceptual load^27^. However, in both studies, “inattentional deafness” was only observed in a small number of trials, suggesting that the assumption of load effects represents an all-or-nothing phenomenon, as described in its strictest form^1^ (“irrelevant information will be excluded from processing”, p.195), cannot be supported.

Regarding early ERP data, the N1 amplitude was not affected by load, and there was no interaction between load and sound volume. Previous working memory load studies found decreased (visual ERPs^8,9^, auditory ERPs^11,12^) or unaffected early ERP amplitudes (visual ERPs^5–7^, auditory ERPs^13^) under high compared to low load. Those heterogeneous results might relate to the differences in the choice of stimuli and their expectancy. For example, two visual studies^17,18^ used emotional faces as distractors and reported load effects on the N170 amplitude. However, several perceptual load studies found no effect of load on the N170 amplitude^37–39^, highlighting the variability of N1/N170 load effects. In the study of Yang et al.^9^, the distractor was predictably presented in the trial, which might possibly enhance suppressive load effects. Several other visual working memory studies^14–16^ found no effect of load on early amplitudes under high vs. low load. For auditory stimuli, results are mixed as well, and findings might depend on analytical parameters, experimental parameters, and statistical power. Simon et al.^12^ could show decreased N1 processing to auditory distractors under high working memory load. The study differs, however, in that the subjects complete a tracking task and are thus subject to a constant executive demand beyond working memory load. Furthermore, the studies by Mahajan et al.^11^ and SanMiguel et al.^13^ raise doubts about the reliability of the occurrence of early effects (see also^4^). Even though both studies use an identical paradigm, Mahajan et al.^11^ reported N1 load effects in contrast to SanMiguel et al.^13^.

Our comparison between heard and missed distractor sounds, regardless of load, showed a modulation of early negativities in the N1 time window in accordance with current theories, suggesting the AAN as a neural correlate of auditory perceptual awareness^28,30^. However, this pattern was only observed for load-independent awareness effects. As described above, there was no effect of load on the auditory ERP in this time window. Therefore, the load effect on detection is not due to typical awareness-related amplitude modulations in the N1 (or AAN) time window but depends on later processes. Thus, load-related modulations of awareness of stimuli are not associated with early NCC in our study.

Regarding late ERP data, there was a main effect of load on the P3 amplitude and an interaction between sound volume and load. The main effect of load on the P3 amplitude is in line with previous studies, which show that high visual working memory load decreased auditory P3 responses^20,22,23,40^. Our P3 effects mirror the results at the behavioral level, where sound detection was decreased under high compared to low load for the medium and high, but not for the low volume sound. Thus, the late neural effect of visual load on auditory stimuli seems to reduce the probability of reported awareness of these stimuli. Our additional analysis also showed a load-independent awareness effect during the P3 stage. The P3 amplitude has been proposed to represent attention, decision-making, and confidence during detection designs^12,28,41–45^. The P3 component does not represent a necessary NCC (in the visual domain^28,44,46^, in the auditory domain^41,47^) but instead seems to reflect the depth of conscious processing, encoding, and thus the degree of reportability of stimuli^41,48^. In our design, it is likely that these processes seem to affect later detection performance and that the working memory task modulates the processing stage associated with the P3 amplitude. Taken together, our data suggest that detection performance might result from a late processing stage, during which load-associated detection effects can be dissociated from load-independent awareness effects.

Referring back to the study of Molloy et al.^17^, which motivated our study, we found similar results at the behavioral level and different results at the neural level. Both studies reported a reduced detection of auditory stimuli under high compared to low (perceptual or working memory) load, associated with a late neural load effect on the P3 amplitude. However, Molloy et al.^17^ additionally found an early neural load effect on the aM100 amplitude, which was absent in our study. In contrast, for ERPs, we conclude that there are no load effects on the N1 based on our study's relatively large sample size, well-controlled stimuli, and many trials per condition. Even if it seems likely that perceptual and working memory load elicit similar effects^6^, it cannot be systematically ruled out that the obtained differences between the two studies could be based on a general difference between the load types. However, other study parameters are more likely to explain these differences. MEG and EEG respond to different dipoles of electromagnetic fields^8,49^, which might explain why MEG reveals effects not seen with EEG data. Ideally, a combination of different neuroscientific methods can resolve this issue. Furthermore, there are differences in the actual study design (besides the different load types). In the study of Molloy et al.^17^, load and distractor stimuli had a simultaneous onset, which represents a critical difference to our working memory load design, where distractor stimuli are presented after load stimuli have already disappeared from the screen. Thus, future studies might directly test whether timing of distractors relative to the load task (encoding, maintenance of information, retention/ decision making) leads to different effects. Moreover, our study compared responses to distractor stimuli against load conditions without distractors to control for general load effects. In other words, without this control, it is difficult to decide whether effects represent effects on distractor processing or effects of task/ targets, especially in cases when targets/ required task responses and distractors overlap.

We would like to note some limitations of our study. Even if the visual task was established as the main task, the instruction to additionally respond to the auditory stimuli created a dual-task-like situation. This was unavoidable for the current design, in which we were interested in both awareness assessments and the examination of ERPs. Therefore, multisensory task-switching effects might have influenced the results^50^ and should be examined in future studies with actual task-irrelevant auditory stimuli. Furthermore, we could only realize two load levels. Future research should increase the range of load levels^38^ to understand better how perceptual load parametrically affects detection and ERP responses. Future studies might also use a broader range of individualized sound levels. Finally, we used a specific operationalization of working memory. Future studies could vary the method and the position of presentation of sounds relative to different phases of the task (encoding, retention, probe). An additional point is that our study used a crossmodal design. While there is no theoretical argument for or against uni- or crossmodal experiments in *Load Theory*^51^, this question needs more empirical research. Future studies with sufficient power might directly compare uni- and multimodal designs. Finally, other analytical strategies such as multivariate pattern analysis^52^ or analysis of specifically predefined electrodes based on prior work might reveal subtle effects in early time windows that were not seen in the current study.

## Conclusion

We investigated the detection and neural processing of auditory stimuli varying in stimulus intensity during a continuous stimuli-unrelated visual load condition alternating between low and high load. We found, depending on stimulus strength, decreased detection and late ERP responses (> 300 ms) to auditory stimuli under high as compared to low load. Findings suggest a late neural effect of visual load on auditory stimuli associated with a reduced probability of reported awareness of these stimuli. This effect is dissociable from general early and late ERP awareness effects.

## Data Availability

The datasets generated and analyzed during the current study are available from the corresponding author on reasonable request.
